# Draft Genome Sequences of *Pseudomonas putida* UV4 and UV4/95, Toluene Dioxygenase-Expressing Producers of *cis*-1,2-Dihydrodiols

**DOI:** 10.1128/genomeA.01419-17

**Published:** 2018-01-04

**Authors:** Timofey Skvortsov, Patrick Hoering, Ksenia Arkhipova, Roger C. Whitehead, Derek R. Boyd, Christopher C. R. Allen

**Affiliations:** a School of Biological Sciences, Queen’s University Belfast, Belfast, United Kingdom; b School of Chemistry, University of Manchester, Manchester, United Kingdom; c School of Chemistry and Chemical Engineering, Queen’s University Belfast, Belfast, United Kingdom

## Abstract

Here, we present draft genome sequences of *Pseudomonas putida* strains UV4 and UV4/95, which demonstrate an ability to conduct a wide range of industrially important biotransformations of arenes, alkenes, and phenols.

## GENOME ANNOUNCEMENT

In a series of pioneering studies, Gibson et al. discovered that *Pseudomonas putida* strains can oxidize certain aromatic hydrocarbons, such as benzene (1) and toluene, producing an enantiomerically pure *cis*-dihydrodiol (2). Further research by Imperial Chemical Industries (ICI) led to the discovery of *P. putida* NCIMB 11767, a toluene dioxygenase-expressing wild-type strain, from which the UV4 mutant was later derived and used in commercial (3) and biotechnological (4, 5) applications.

Two isolates of the *P. putida* UV4 strain were used for genome sequencing in this study, *P. putida* UV4, which was acquired from Stephen Taylor (ICI Biological Products) in 1984 and has been continuously cultured in the laboratory since that time, and *P. putida* UV4/95, which was subsampled from the main culture of *P. putida* UV4 in 1995 and preserved as a lyophilized sample until being revived for sequencing in 2015.

Bacterial cell cultures were grown overnight in LB medium at 30°C with 140-rpm shaking. Genomic DNA was extracted using the PowerSoil DNA isolation kit (Mo Bio, Inc., Carlsbad, CA, USA). Whole-genome sequencing was performed at MR DNA (Shallowater, TX, USA) using the Illumina MiSeq platform (2 × 150 bp), and sequence reads were assembled using NGen DNA assembly software (DNAStar, Inc., Madison, WI, USA) at the site. The assembly produced 31 contigs for UV4 and 34 contigs for UV4/95 with an average coverage of 40×, and an assessment with CheckM (6) characterized the draft genomes as 100% complete. The assemblies were annotated online using the NCBI Prokaryotic Genome Annotation Pipeline (PGAP) (7).

The draft genomes of both isolates have a GC content of 61.6%, with UV4/95 being longer (6.2 Mb versus 6.1 Mb for UV4). The total number of genes in UV4 was 5,703, while 5,790 genes were detected in UV4/95. Application of the species identification tool SpecI (8) identified *P. putida* F1 (1) as the closest related strain. A comparative analysis of the UV4 and UV4/95 draft genomes with *P. putida* F1 using Mauve (9) revealed the absence of a 90-kb-long region in UV4 and F1 compared to UV4/95, which was later identified as a genomic island by IslandViewer (10). The genomic island contains 88 genes, the majority of which are of unknown function or related to phage proteins.

A detailed functional annotation of protein-coding genes with RASTtk (11) assigned 35% of them to functional categories of SEED subsystems. A number of genes involved in the functionalization of aromatic compounds were detected, with dioxygenases and monooxygenases among them. Analysis with antiSMASH version 4.0 (12) predicted 40 biosynthetic clusters in UV4/95 and 42 in UV4.

*P. putida* strain UV4 has substantial enzymatic potential and is widely used in biotechnological applications. The reconstruction of its genomic sequence significantly improves our understanding of its physiological and biochemical peculiarities and provides new options for genetic manipulation of this microorganism in industrial and academic settings.

### Accession number(s).

The annotated draft genome sequences of *P. putida* UV4 and UV4/95 have been deposited at NCBI GenBank under the accession numbers NHBB00000000 and NHBC00000000, respectively.
